# Combining Peyton’s four-step approach and Gagne’s instructional model in teaching slit-lamp examination

**DOI:** 10.1007/s40037-014-0136-x

**Published:** 2014-10-08

**Authors:** Jia Yu Ng

**Affiliations:** Department of Ophthalmology, University Hospital Ayr, Dalmellington Road, Ayr, Scotland, UK

**Keywords:** Gagne’s instructional model, Slit lamp teaching, Peyton steps, Lesson planning

## Abstract

Developing skills in performing basic slit-lamp biomicroscopy is an important element of the ophthalmology undergraduate curriculum. As a doctor working in an ophthalmology department, I often provide slit-lamp teaching for medical students. This paper describes a lesson plan for this technique using Gagne’s nine events of instruction. The presented lesson plan is a combination of Gagne’s nine events of instruction and Peyton’s four-step approach. Gagne’s nine events of instruction correlate with and address the mental conditions of learning when adult learners are presented with various stimuli. Peyton’s four-step approach is a model for teaching practical skills that consists of demonstration, deconstruction, explanation, and performance. This article describes a slit-lamp biomicroscopy teaching session using Gagne’s nine events of instruction. Each step is carefully elaborated with relevant activities to suit learners with various learning styles. Peyton’s approach is used to teach the actual skill. This lesson plan is particularly relevant for tutors designing slit-lamp biomicroscopy teaching for undergraduate students, foundation doctors, general practitioners and emergency department staff. Ultimately, this lesson plan also serves as a model that is applicable for acquiring many other practical skills. The flexible adoption of Gagne’s nine events of instruction in combination with other teaching models helps in the planning of effective teaching sessions.

## Introduction

The basic slit-lamp biomicroscopy examination is an ophthalmological technique to inspect the anterior segment of the eye. The technique is difficult to master, yet it is an important element of the ophthalmology undergraduate curriculum. As a doctor working in an ophthalmology department, I often plan and deliver slit-lamp biomicroscopy teaching sessions for undergraduate medical students. In this article, using Gagne’s nine events of instruction I describe a lesson plan for a two-hour slit-lamp biomicroscopy session; Peyton’s four steps are used for teaching the practical skills (demonstration, deconstruction, explanation, and performance).

One of the most important considerations while using Gagne’s nine events of instruction is to identify the type of learning outcomes to be achieved [1]. Of the learning outcomes, the skills needed for slit-lamp biomicroscopy examination most clearly correspond to motor skills. This lesson plan can be easily applied to other practical skills teaching sessions.

Prior to a slit-lamp biomicroscopy session, I encourage students to revise the following areas in preparation for the session:

- Basic anatomy of the eye
- Indication for slit-lamp biomicroscopy examination
- Know the common pathologies of the anterior segment of the eye.

Starting from the above prerequisites, students would be able to carry out slit-lamp examination purposefully. Gagne’s nine events of instruction correlate to and address the mental conditions of learning. The following illustrates a variety of strategies for each of Gagne’s events, tailored to accommodate various learning styles.

## Gaining attention

To enhance learning, I gain students’ attention to stimulate their interest and motivate learning. This is done by:

- An abrupt auditory stimulus as opening: i.e. a two-second blast of loud music to bring attention.
- Socratic method of exploratory questioning is an effective method of introducing a new topic to the audience [2]. This is done by posing thought-provoking questions such as ‘when would you use a slit-lamp instead of an ophthalmoscope?’
- Projecting a three-minute video showing slit-lamp biomicroscopy being used in real time.

This method simultaneously targets visual, auditory and kinaesthetic learning styles [3, 4].

## Informing learners of objectives

To make sure students have the same aim for the session as planned, learning objectives are discussed at this early stage. Objectives should be clear and well-defined realistic expectations that are measurable and achievable for the session. For example, the pre-determined objectives for this session are:

Upon completing this session, you will be able to:

- Operate a slit-lamp biomicroscope
- Examine the anterior segment of the eye with a slit-lamp biomicroscope.

To aid a meaningful learning experience, I discuss these learning objectives with the students at this stage to ensure that they agree and understand why they are learning this [5]. For example, revealing the objectives sequentially enhances the opportunity to clarify each item before continuing to the next. Student objectives may sometimes be different, for example they may wish to know just enough to pass their exam. Learners also have the opportunity to point out their pre-instruction competences, which could then be omitted, or areas of difficulties that would need special attention [5]. I then have the opportunity to tailor the session to accommodate joint objectives accordingly if needed.

## Stimulate recall of prior learning

According to constructivist learning theory, adults construct their knowledge based on connections with prior learning and experiences [6]. Interactive group discussion on previous attempts at using a slit-lamp biomicroscope, and experience from workplace observations (i.e. general practice and optometry), could facilitate such recall. To further augment this process, I ask students about the function and context in which slit-lamp biomicroscopy is used. A plastic model of the eye is then used to recap the anatomy of the anterior segments.

These activities aim to motivate learners to build more detailed knowledge upon a basic pre-existing foundation. Indeed, developing understanding in this systematic fashion particularly suits a constructivist approach. Moreover, this method also complements multi-intelligence theories as its variety of memory aids engage visual, linguistic, kinaesthetic, intrapersonal, and interpersonal intelligences [7].

## Presenting the stimulus

The detailed stages involved in slit-lamp biomicroscopy examination are explained using a PowerPoint presentation. A flowchart that summarises the steps is also provided as a hand-out. Furthermore, examples of pathologies that occur in each anatomical structure are given in the lecture as we go along. This will help students make sense of why each particular step is carried out. Thereafter, I incorporate Peyton steps to teach the actual procedural skills; as such, the first stage consists of my demonstrating each element of an slit-lamp biomicroscopy examination at normal speed without any explanation [8]. These activities stimulate visual-audio, linguistic, as well as interpersonal intelligences; [7] in addition, the flowchart also appeals to visual learners.

## Providing learning guidance

To begin interactive learning, I elicit the names of different components of the slit-lamp and their functions. Following this, I deconstruct the examination by repeating the slit-lamp biomicroscopy steps with a full explanation, describing all the necessary sub-steps and offering practical tips (Peyton Step 2) [8]. Students are encouraged to ask questions at this stage. This is followed by a comprehension phase where the students take the lead in explaining each sub-step of the slit-lamp biomicroscopy examination with the teacher following their instructions (Peyton Step 3) [8]. This again stimulates linguistic and kinaesthetic learning styles. Having students verbalise the step-by-step sequence also allows me to check their understanding as we go along.

## Eliciting performance

I allocate the greater proportion of time for this step as it allows learners the opportunity to confirm their learning through performing. This step is equivalent to Peyton Step 4 where the student attempts to complete a slit-lamp biomicroscopy examination autonomously [8]. In subgroups of two or three, students familiarise themselves with the slit lamp, taking turns to perform slit-lamp biomicroscopy examinations on each other’s eyes under careful supervision. By doing so, I am ensuring that a stimulating range of classroom activities are used to enhance the teaching and learning process.

## Providing feedback

While learners are performing the slit-lamp examination on their peers, I provide individual guidance and immediate in situ feedback. Learners are encouraged to clarify queries as they arise. In addition, volunteering peers are invited to comment from their simulated patient perspective, e.g. whether the chin rest position is at a comfortable height, whether the bright lights are causing discomfort, etc. An appropriate feedback model—such as an agenda-led outcome-based analysis (ALOBA model)—should be used to provide feedback [9].

## Assessing performance

Following the guided hands-on practice, students have their slit-lamp biomicroscopy examination skills assessed. They will now switch partners and demonstrate the whole procedure without help or guidance. I use a combination of checklists and patient (volunteering peers) feedback for formative assessment. This is then compared with the student’s self-evaluation in order to identify further learning points. This stage in the process involves visual, logical, linguistic, kinaesthetic, and interpersonal intelligences [4, 7].

## Enhancing retention and transfer

Following the formative assessment outlined above, students are given the opportunity to examine the eyes of volunteer patients. This allows them to make sense of the learning event and thereby enhance their learning. It also helps them to transfer the newly acquired technique to clinical practice [1].

Thereafter, the session is concluded by reviewing the learning objectives achieved, answering any remaining questions, eliciting learner evaluations of the session, and suggesting post-session reading material [10]. Subsequently, students have the opportunity to perform more slit-lamp examinations throughout their clinical attachment in the department; indeed, they are encouraged to perform Mini-Clinical Evaluation Exercises in the clinic for further evaluation. These activities stimulate the logical, linguistic, kinaesthetic, and interpersonal intelligences of our learners [7].

Using Gagne’s nine events of instruction to teach slit-lamp skills has repeatedly received positive feedback from the medical students in our unit. Our online student evaluation exercises have shown that all students feel that their confidence in using slit lamps increased following the session, with 50 % of them *strongly* agreeing with this sentiment.

For the teacher, Gagne’s nine events of instruction provide a checklist for structuring the teaching and learning activities on an effective session plan. The steps are clearly illustrated which help to establish whether each successive goal has been achieved. It also allows for flexibility in allocating time to each step – i.e., allowing you to move on when appropriate for your teaching context. Admittedly, sometimes I feel that I am simply ‘filling in the blanks’ while planning for such sessions, particularly when implementing Peyton’s four steps (which I have spread across Gagne’s steps four to six).

## Conclusion

Gagne’s nine steps provide an effective and systematic learning programme as they give structure to the lesson plan that suits a variety of learning styles. The majority of students possess multimodal learning styles and hence learn most effectively from a blend of activities that stimulate the visual, aural, verbal (read-write) and kinaesthetic sensory modalities [3]. Ultimately, this lesson plan also serves as a model that is applicable for acquiring many other practical skills.
